# The emerging view on the roles of butyrate in *Clostridioides difficile* pathogenesis

**DOI:** 10.1128/iai.00047-25

**Published:** 2025-09-24

**Authors:** Horia A. Dobrila, Andrew J. Hryckowian

**Affiliations:** 1 Department of Medicine, Division of Gastroenterology and Hepatology, University of Wisconsin-Madison School of Medicine and Public Health 5232, Madison, Wisconsin, USA; 2 Department of Medical Microbiology & Immunology, University of Wisconsin-Madison School of Medicine and Public Health 5232, Madison, Wisconsin, USA; 3 Microbiology Doctoral Training Program, University of Wisconsin-Madison 5228, Madison, Wisconsin, USA; University of California at Santa Cruz, Santa Cruz, California, USA

**Keywords:** *Clostridium difficile*, pathogenesis, virulence regulation, gut microbiome, metabolism, sporulation, toxins

## Abstract

The Centers for Disease Control and Prevention classifies *Clostridioides difficile* as an urgent threat to the nation’s health, as it causes 450,000 infections, 15,000 deaths, and 1 billion dollars in excess healthcare costs per year in the United States. Current treatments for *C. difficile* infections (CDIs) are antibiotics and, in recurrent cases, microbiome restoration therapy (MRT). Antibiotics contribute to antibiotic resistance and recurrent CDIs. Although MRTs (e.g., defined consortia of microbes or fecal transplant) are increasingly accessible, the long-term sustainability and accessibility of these treatments remain to be determined. These limitations highlight the need for more precise strategies for coping with CDI. Because a disrupted (dysbiotic) gut microbiome is the primary risk factor for CDI, a better understanding of the interactions between *C. difficile*, the microbiome, and the host will aid the development of such treatments. Butyrate is a prominent microbiome-host co-metabolite that is influenced by host dietary fiber intake and differentiates healthy from dysbiotic gut ecosystems. Emerging evidence supports that butyrate is a key determinant of *C. difficile* fitness and pathogenesis. Here, we review the current literature and gaps in knowledge about how butyrate-rich gut environments exclude *C. difficile*, and how butyrate impacts *C. difficile* growth, metabolism, toxin production/release, and sporulation. We further discuss the implications of continued study of butyrate’s impacts on CDI, including the eventual development of new strategies to mitigate CDI in at-risk human populations.

## *CLOSTRIDIOIDES DIFFICILE* IS A SERIOUS HEALTHCARE CONCERN

The Centers for Disease Control and Prevention classifies *Clostridioides difficile* as an urgent threat to the nation’s health, as it causes 450,000 infections, 15,000 deaths, and 1 billion dollars in excess healthcare costs per year in the United States (1, 2). Most *C. difficile* infections (CDIs) occur in healthcare settings, where CDI is the most common cause of infectious diarrhea (3). Known and suspected risk factors for CDI include antibiotics, proton pump inhibitors, impaired immune function, advanced age, and diet, all of which are associated with dysbiotic gastrointestinal (GI) microbiomes (4–6). Though most CDIs are associated with antibiotic treatment, 22% of individuals with community-acquired CDI have no recent history of antibiotic use. Factors affecting persistent and recurrent CDIs remain poorly defined (7, 8). Despite the morbidity and mortality caused by *C. difficile*, up to 15% of healthy adults are asymptomatic carriers of toxigenic *C. difficile* (9), highlighting the gaps in our understanding of *C. difficile*.

*C. difficile* pathogenesis is mediated by secreted protein toxins. The two main toxins produced by *C. difficile* are TcdA and TcdB. *C. difficile* releases these toxins into the gut lumen. They are internalized into colonocytes and inactivate Rho GTPases, which leads to disruption of the actin cytoskeleton and tight junctions. Toxin-induced cellular damage causes cell lysis, inflammation, and a weakened epithelial barrier, which lead to the diarrhea characteristic of CDI (10–13). *tcdA* and *tcdB* are encoded within a 19.6 kb pathogenicity locus (14), which also contains three accessory genes involved in toxin regulation and secretion. Specifically, *tcdR* encodes the alternative sigma factor responsible for activating the expression of toxin genes (15), *tcdC* encodes the anti-sigma factor that inhibits TcdR (16), and *tcdE* encodes a holin-like protein that forms pores in the *C. difficile* cell membrane and is involved in toxin secretion (17, 18). Some “hypervirulent” *C. difficile* strains produce a third secreted toxin, CDT, which is internalized into colonocytes and catalyzes ADP ribosylation; this activity disrupts the actin cytoskeleton and contributes to CDI pathogenesis (19–21). Emerging evidence suggests that *C. difficile* toxin-mediated inflammation benefits *C. difficile* by allowing it to acquire host nutrients (22, 23) and outcompete inflammation-sensitive gut microbes (22–25).

Another key feature of *C. difficile* biology is its ability to form spores, likely in response to stress and nutrient limitation. Sporulation in *C. difficile* has been tied to its metabolic environment, particularly with emphasis on peptides (26), carbon catabolite availability (27, 28), and branched amino acids present in its environment (29). Genetic regulation of *C. difficile* sporulation is tightly controlled (26, 30, 31). Through a complex series of morphological and biochemical changes, sporulating *C. difficile* cells divide asymmetrically and form an environmentally resistant spore, which is released from the larger mother cell by cell lysis. Spores are highly resistant to environmental stresses such as heat, chemicals, desiccation, oxygen, and ethanol-based sanitizers (32). These spores allow *C. difficile* to survive outside the GI tract and transmit to new hosts, where they germinate to form vegetative cells (33).

## THE MICROBIAL AND METABOLIC MILIEU OF CDI

Dysbiosis is the primary risk factor for CDI, and several microbial taxa directly impact *C. difficile* fitness (34–36). However, inter-individual variation in gut microbiome composition complicates definitions of CDI susceptibility and resistance. Human studies and animal models suggest that microbiome-dependent metabolites, rather than microbiome composition, define CDI susceptibility and resistance (37–40). Therefore, a focus on metabolites may allow for more readily translatable findings.

The gut contains thousands of diverse molecules derived from the diet and host/microbiome metabolism. Many of these impact *C. difficile* fitness and pathogenesis. For example, bile acids, metals, amino acids, sugars, organic acids, and short-chain fatty acids (SCFAs) affect *C. difficile in vitro* and in animal models (22, 23, 35–37, 41–46). SCFAs (in particular, acetate, propionate, and butyrate) are major metabolic products of microbiome metabolism and are primarily generated by microbial degradation of dietary fiber (47, 48). Collectively, SCFAs reach concentrations >100 mM in the colon and are the most concentrated metabolites in the human distal gut (49). SCFAs are influenced by microbiome composition, diet, antibiotics, and inflammation (50). Most previous research on SCFAs has focused on their impacts on the host. For example, SCFAs are metabolized by colonocytes or are transported systemically via portal circulation (47). SCFAs also enhance gut barrier function and modulate immune responses (47, 48). SCFA deficiencies are associated with inflammatory bowel disease and increased susceptibility to pathogens (25). Because SCFAs are so pivotal in host-microbiome cross-talk, microbiome studies often equate a “healthy gut” with high SCFAs and a “dysbiotic gut” with low SCFAs (25).

Of the SCFAs produced in the GI tract, butyrate stands out as an impactful microbiome-host co-metabolite. Microbiome-produced butyrate is the main source of energy for gut colonocytes (51, 52), where it is taken up and consumed by colonocytes through fatty acid β-oxidation, a process that consumes oxygen and supports the growth of obligate anaerobes. When butyrate is limited, colonocytes switch from fatty acid β-oxidation to glycolysis. Glycolysis does not consume oxygen and subsequently contributes to dysbiosis (53, 54). Additionally, butyrate promotes the integrity and maintenance of gut barrier function by increasing the number of tight junctions between colonocytes (55), increasing expression of Mucin 2 to strengthen the mucus layer of the gut (56), and by enhancing HIF-1 activation in colonocytes (57). Additionally, butyrate has direct immunomodulatory effects. It suppresses proinflammatory effectors in macrophages within the lamina propria (58), as well as the differentiation of dendritic cells from bone marrow stem cells (59) through histone deacetylase inhibition.

In addition to its direct effects on the host, animal and human data show that high butyrate characterizes a gut that is non-permissive to CDI (37, 45, 60, 61). *In vitro*, butyrate inhibits *C. difficile* growth, increases sporulation, and leads to elevated *C. difficile* toxins in culture supernatants (37, 45, 46, 57, 60, 62–64). Additionally, exogenous butyrate is internalized into *C. difficile* cells and is incorporated into its butyrogenic metabolic pathways, potentially contributing to these phenotypes. The conclusion that butyrate is associated with non-permissive environments for *C. difficile in vivo* while also enhancing *C. difficile’s* virulence mechanisms *in vitro* seems contradictory. However, taken together, these data on CDI permissiveness, growth, sporulation, and toxin expression/release suggest that *C. difficile* senses butyrate as a signal of a competitive environment and adjusts its virulence to maintain a dysbiosis-associated niche or to transmit to new hosts (25). Therefore, current data suggest that butyrate is a context-dependent regulator of *C. difficile* fitness and virulence traits. As such, butyrate stands out as an impactful target to better understand microbiome-host co-metabolism and to improve therapies against *C. difficile*.

## THE IMPACTS OF FIBER CONSUMPTION AND GI BUTYRATE ON CDI IN MICE AND HUMANS

A growing body of literature suggests that fiber-deficient diets favor infection by bacterial pathogens such as *C. difficile* in humans and in animal models of CDI (37, 45, 65–68). We previously showed that fiber-deficient rodent diets (which recapitulate fiber deficiencies of human Western diets) perpetuate CDI in mice. In contrast, mice fed high-fiber diets containing a complex mixture of fiber types (standard rodent chow) (37) or diets containing isolated fiber types that elevate GI butyrate (e.g., inulin or resistant maltodextrin) (45) clear *C. difficile* within days. Similarly, dietary xanthan gum leads to rapid clearance of *C. difficile* from the mouse gut and is associated with elevated GI butyrate levels (69). In contrast, fiber types that do not elevate GI butyrate (e.g., fructooligosaccharides and gum Arabic) do not suppress *C. difficile* burdens (45). These data suggest that CDI suppression is not generalizable to all fibers, but rather to a fiber’s butyrogenic capacity. To date, eight diets containing various fiber types confirm this link (45, 69), but it is unclear whether the effects are caused by butyrate or are simply associated with elevated butyrate (70).

Additional connections between butyrate and *C. difficile* fitness have been drawn using mouse and hamster models of CDI, without manipulating dietary fiber. In a Syrian hamster model of CDI, both *C. difficile* colonization and symptomatic CDI differed between infant vs adult animals, where the development of the hamster gut microbiome and subsequent production of SCFAs were correlated with these observations. From this study, only GI butyrate levels in the hamster reached inhibitory levels for *C. difficile*, indicating butyrate’s importance during experimental infection (60). In another study using gnotobiotic mice colonized with a butyrate producer (*Clostridium sardiniense*), mice succumbed more rapidly to CDI than gnotobiotic mice colonized with an amino acid fermenter (*Paraclostridium bifermentans*), highlighting context-dependent effects of butyrate on CDI (71). Finally, SCFAs have beneficial pleiotropic effects on host biology, but the impacts of SCFAs on the host as they relate to CDI outcomes are mostly unexplored. One study treated mice with butyrate and showed that they had less severe CDI but did not measure *C. difficile* burdens (70). These effects were mediated through farnesoid X receptor, which is activated by bile acids, but not by butyrate (70). Also, rather than treating with tributyrin, a prodrug of butyrate that efficiently delivers butyrate to the distal GI (72, 73), mice were given butyrate by oral gavage, which is absorbed by the proximal GI (74). So, it is unclear if the changes in this study were due to systemic effects of butyrate on the immune system. Another study administered both butyrate and tributyrin to mice infected with *C. difficile* and found that while CDI severity was lessened *in vivo*, butyrate/tributyrin did not impact colonization resistance against *C. difficile* or its toxin production (using both germ-free mice and conventional mice) (57). These findings suggest context-dependent differences *in vivo*, which can drastically change how butyrate impacts CDI, emphasizing that more knowledge of the direct effects and relative impacts of fiber and butyrate on *C. difficile* fitness and virulence phenotypes during CDI is needed.

There are similar links between fiber, butyrate, and CDI in humans. We showed that CDI patients have lower butyrate (but not acetate or propionate) in their stool than healthy controls (45). Others showed that CDI patients have lower propionate and butyrate (but not acetate) than healthy controls (70), and GI butyrate is positively associated with successful fecal transplant for recurrent CDI (61). Also, fiber consumption is associated with lower odds of *C. difficile* colonization in humans and a concomitant increase in GI SCFAs (67, 68). Therefore, fiber consumption and high GI butyrate are associated with lower CDI risk in humans.

Diet has a much broader impact than simply modulating GI butyrate levels. Shifts in diet lead to large-scale changes to gut microbiome membership (37), changes in host metabolism (51, 52), and impact other parameters such as stool consistency and gut transit time, all indicating that the effects of butyrogenic fiber on CDI are also impacted by additional variables other than butyrate. Since existing *in vivo* work occurred against the backdrop of the complex microbial and metabolic milieus of the GI tract, a more granular understanding of butyrate production in the gut and its direct impacts on *C. difficile* is needed.

## THE EFFECTS OF EXOGENOUS BUTYRATE ON *C. DIFFICILE* GROWTH AND METABOLISM

Butyrate inhibits the growth of diverse *C. difficile* isolates *in vitro* (37, 45, 46, 57, 60, 62, 63). The presence and magnitude of butyrate-dependent growth inhibition vary based on concentrations of butyrate and other nutrients in the media (Table 1). For example, while several studies identified a dose-dependent inhibition of growth with butyrate supplementation, others were unable to replicate those findings. In one study, the addition of various sugar sources, such as cellobiose, maltose, and trehalose, abolished the butyrate-dependent growth defect, and fructose, mannose, and mannitol supplementation induced a butyrate-dependent increase in growth (63). This indicates that differences in nutrient availability drive differences in metabolic flux or preferences for different metabolic pathways that may influence butyrate’s inhibitory effects on *C. difficile*. Additionally, a study from our group identified that in a basal-defined medium (BDM), *C. difficile* does not produce its own butyrate and does not experience a butyrate-dependent growth defect (46), further emphasizing that metabolic flux through butyrogenic pathways is important for this phenotype. Supportively, when supplied with exogenous butyrate, *C. difficile* produces less of its own butyrate. To address the underlying effects of exogenous butyrate on *C. difficile* butyrogenic metabolism, we supplemented cultures with ^13^C_4_-butyrate. We found that ^13^C_4_-butyrate is incorporated into intracellular CoA pools where it is metabolized in an energetically unfavorable direction to crotonyl-CoA and hydroxybutyryl-CoA (46). This demonstrates two important concepts. First, exogenous butyrate is internalized into *C. difficile* cells and alters *C. difficile* metabolism. Second, butyrate-dependent growth defects only occur when *C. difficile* is producing butyrate as a metabolic end product.

**TABLE 1:** Previous studies that demonstrated butyrate-dependent growth inhibition in *C. difficile in vitro*

Reference	Media	*C. difficile* strains	Butyrate concentrations	General findings
(60)	Brain-heart infusion (BHI)	TTU 614	1.5 μeq/mL 3.1 μeq/mL 6.2 μeq/mL 12.5 μeq/mL 25 μeq/mL 50 μeq/mL 100 μeq/mL	The MIC of butyrate is 6.2 μeq/mL at pH 6, 12.5 μeq/mL at pH 6.2, and 25 μeq/mL from pH 6.4–7.
(62)	BHI	1551 2165 2167 NAP1/027	25 mM 50 mM 75 mM 100 mM	Butyrate inhibits *C. difficile* growth at 50 mM and greater. No dose-dependent effect observed.
(37)	Cartman’s *C. difficile* minimal medium (CDMM) without glucose	630	10 mM 30 mM	Butyrate decreases *C. difficile* growth rate in a dose-dependent manner.
(57)	BHI	VPI 10463	1 mM 10 mM 50 mM	Butyrate negatively impacts *C. difficile* growth at 50 mM.
(45)	Modified reinforced clostridial medium (mRCM)	630 BI-9 CD196 CD305 CF5 Liv022 Liv024 M120 M68 R20291 TL174 TL176 TL178	6.25 mM 12.5 mM 25 mM 50 mM	Butyrate has dose-dependent impacts on *C. difficile* growth rate and lag time across 13 genetically diverse strains.
(63)	BHI Karasawa’s CDMM (supplemented with various sugars)	630 VPI 10463 R20291	5 mM 10 mM 25 mM 50 mM	In BHI, butyrate has a dose-dependent growth defect in BHI in *C. difficile* 630, but not in VPI 10463 or R20291. Supplementation of various sugars in CDMM modulates the butyrate-dependent growth defect: Medium supplemented with lactose and raffinose maintained the growth defect Medium supplemented with cellobiose, maltose, and trehalose had no butyrate-dependent growth defect Medium supplemented with fructose, mannose, and mannitol had butyrate-dependent growth enhancement.
(46)	mRCM (+mannose or +glycine) Glucose-free BDM supplemented with added nutrients (+2% casamino acids, +2× amino acids, and +glucose)	630	50 mM	A butyrate-dependent growth defect is observed in 630 in an undefined complex medium (mRCM) but not in a defined minimal medium (BDM). The butyrate-dependent growth defect increases in conjunction with nutrient availability. The butyrate-dependent growth defect occurs in a medium where *C. difficile* produces its own butyrate, and *C. difficile* produces less butyrate when exogenous butyrate is present. Supplementation with ^13^C_4_-butyrate revealed that butyrate is internalized into CoA pools and forces butyrogenic metabolic pathways in a reverse, metabolically unfavorable, direction.

However, butyrate does not inhibit the growth of all bacteria (75, 76). One study on *Bacteroides* identified that while butyrate can have an inhibitory effect, supplementation with different sugar sources can modulate and even negate this effect depending on the strain used (76). Therefore, different gut microbes differentially sense and respond to butyrate, possibly highlighting important differences in their metabolic capabilities and their niches within the gut. It is reasonable to assume that organisms that are negatively affected by butyrate respond to butyrate-rich environments by altering their gene expression. In the case of a pathogen like *C. difficile*, it is likely that these responses involve the expression of genes involved in virulence.

## BUTYRATE-DEPENDENT EFFECTS ON *C. DIFFICILE* TOXINS

During dysbiosis, *C. difficile* can access nutrients otherwise consumed by the microbiome, and it proliferates. However, as the microbiome recovers from dysbiosis, *C. difficile* must compete with these microbes for nutrients (24). *C. difficile* can leverage the inflammation created by its toxins to enhance its infection by both suppressing the growth and recovery of other gut microbes (23, 77) and liberating host metabolites (23) that provide this pathogen with a competitive advantage. Supportively, *C. difficile* toxin expression and release are directly tied to multiple features of a competitive gut environment (e.g., low proline, branched-chain amino acids, fructose-1,6-bisphosphate, or cysteine; and high autoinducing peptides or butyrate) (78). This highlights that the exquisite control *C. difficile* has over its virulence. We and others showed that butyrate leads to increases in *C. difficile* toxins in culture supernatants in a dose-dependent fashion (37, 46, 57, 63, 64). As observed for butyrate-dependent growth inhibition in *C. difficile*, the presence and magnitude of butyrate-dependent toxin expression and release vary based on the concentrations of butyrate and other nutrients in the media (Table 2). Nonetheless, the consensus from these studies confirms that butyrate-induced toxin production/release occurs across diverse *C. difficile* strains and under different growth conditions, emphasizing the generalizability of the phenotype.

**TABLE 2:** Previous studies that demonstrated butyrate-dependent effects on *C. difficile* toxins *in vitro*

Reference	Media	*C. difficile* strains	Butyrate concentrations	General findings
(64)	Peptone-yeast (PY) ± 10 mM cysteine PY glucose supplemented defined medium (SDM)	VPI 10463	30 mM	Butyrate increases detectable toxin yield across all growth media tested. Butanol decreases toxin yield in a dose-dependent manner in PY.
(37)	Cartman’s *C. difficile* minimal medium (-glucose)	630	10 mM 30 mM	Butyrate increases TcdB in culture supernatants in a dose-dependent fashion.
(57)	Brain-heart infusion (BHI)	VPI 10463	1 mM 10 mM 50 mM	Butyrate (10 mM and 50 mM) increases TcdA and TcdB in culture supernatants.
(63)	BHI	630 VPI 10463 R20291	5 mM 10 mM 25 mM 50 mM	Butyrate increases detectable toxins in *C. difficile* 630, VPI 10463, and R20291. 25 mM butyrate induces more toxin than 5 mM butyrate, but a significant dose-dependent response was not identified with an expanded range of concentrations. Elevated toxin levels are detectable as early as 6–12 h post-inoculation. Small but significant upregulation of *tcdR* (and small but significant downregulation of *tcdC*) in the presence of 25 mM butyrate at early and late log phase growth. Small but significant upregulation of *tcdA* in the presence of 25 mM butyrate at early log phase growth.
(46)	Modified reinforced clostridial medium	630 R20291	50 mM	Butyrate positively impacts toxin levels in culture supernatants at 48 h post-inoculation. Butyrate does not strongly impact toxin transcripts (*tcdA* or *tcdB*) in log phase or stationary phase growth. Butyrate positively affects transcripts of *tcdE*, *cwp19*, and CD630_21840 during the stationary phase. Butyrate positively impacts extracellular lactate dehydrogenase at 48 h post-inoculation, leading to the hypothesis that butyrate impacts toxin release (potentially via autolysis).

Two recent studies interrogated butyrate-dependent expression of toxin genes and toxin release machinery. In our work, RNA-seq revealed that butyrate does not strongly impact *tcdA/tcdB* transcription in *C. difficile*. While *tcdA*/*tcdB* transcripts are slightly elevated during log phase growth with butyrate, these increases are less than a twofold increase (log_2_ fold change <1), and therefore, these genes were deemed not strongly differentially expressed in our work. Instead, *tcdE*, CD630_21840 (a putative endolysin that facilitates toxin release independent of autolysis, potentially in conjunction with TcdE) (79), and *cwp19* (a lytic transglycosylase involved in stationary phase autolysis) (80) are all strongly upregulated (log_2_ fold change >1, adjusted *P*-value < 0.05) during the stationary phase, coinciding with the high toxin levels in culture supernatants. Cwp19-mediated autolysis and TcdE-mediated secretion are co-existing mechanisms for toxin release (80). Supportively, butyrate exposure leads to increased autolysis of *C. difficile* cells (46). These data suggest that butyrate affects TcdE-dependent secretion and Cwp19-dependent autolysis, which together contribute to butyrate-dependent toxin release. However, it is unclear if toxin release via autolysis and TcdE occurs on different time scales or due to different signals in culture and during CDI. Another study that grew *C. difficile* in a different growth medium (brain-heart infusion [BHI]) showed butyrate-dependent increases in toxin levels in culture supernatants. This corresponded to small but significant increases in *tcdR* and *tcdA* transcripts and small but significant decreases in *tcdC* transcripts in the presence of butyrate (63). Taken together, these data support that different metabolic landscapes may differentially impact butyrate-dependent toxin expression and release.

## BUTYRATE IMPACTS *C. DIFFICILE* SPORULATION

Spores are the sole vehicle of *C. difficile* transmission. While sporulation has been previously tied to metabolite availability, butyrate-responsive sporulation is a newly appreciated facet of *C. difficile* biology that ties *C. difficile* transmission to yet another aspect of the metabolic activity of the gut microbiome (46, 63) (Table 3).

**TABLE 3:** Previous studies that demonstrated butyrate-dependent sporulation in *C. difficile in vitro*

Reference	Media	*C. difficile* strains	Butyrate concentration(s)	General findings
(63)	Brain-heart infusion 70:30	630	5 mM 10 mM 25 mM 50 mM	Butyrate-dependent sporulation occurs in *C. difficile* when grown in BHI containing 25 mM and 50 mM butyrate, as determined by quantifying germination-proficient spores. Butyrate-dependent sporulation occurs in *C. difficile* when grown in 70:30 medium containing 25 mM butyrate, as determined by phase contrast microscopy. Numerous sporulation genes are upregulated in the presence of 25 mM butyrate in BHI (e.g., stage II, III, IV, and V sporulation genes and sporulation sigma factors E, F, and G).
(46)	Modified reinforced clostridial medium 70:30	630 R20291	50 mM	Numerous sporulation genes are upregulated in 630 in the presence of 50 mM butyrate in mRCM (e.g., stages II, III, IV, and V sporulation genes and sporulation sigma factors E, F, G, and K). Butyrate-dependent sporulation occurs in 630 and R20291, as determined by quantifying germination-proficient spores. Butyrate does not increase viable spore counts in 630 grown in 70:30 medium.

While the mechanisms underlying butyrate-dependent sporulation in *C. difficile* are unknown, this new literature, combined with previous work in *C. difficile* and relatives, provides impactful starting points. Notably, in both *C. difficile* and *Bacillus subtilis*, sporulation initiation is controlled by phosphorylation of the master regulator, Spo0A. Once phosphorylated, Spo0A is responsible for inducing expression of numerous genes, including the early-stage sporulation sigma factors, SigE and SigF. These early-stage sporulation sigma factors then activate transcription of the genes encoding late-stage sporulation sigma factors SigG and SigK, in a hierarchical cascade of gene expression to carry out this complex biological process (81). RNA-seq from two independent studies showed that the most prominent class of genes upregulated in response to butyrate is “sporulation.” Specifically, in one study by Baldassare et al., butyrate induced expression of sporulation sigma factors such as SigE, SigF, SigG, as well as other stages II, III, IV, and V sporulation genes and endopeptidases (63). This group also identified that genes encoding many of the known regulators of virulence and sporulation, such as CcpA, Rex, PrdR, and CodY, were not significantly differentially expressed in the presence of butyrate. Supportively, in our work, butyrate also induced expression of genes encoding sporulation sigma factors, SigE, SigF, SigG, and SigK, and numerous stages II, III, IV, and V sporulation genes and additional spore-specific genes (46). In contrast to the paper by Baldassare et al., our work showed significant (adjusted *P*-value < 0.05) downregulation of *ccpA, rex, prdR*, and *codY*; however, these were small changes in expression (and log_2_-fold change <2 and >−2) and were therefore not reported as differentially regulated. Upregulation of the downstream sporulation sigma factors but not *spo0A* during log-phase growth suggests that butyrate could be directly or indirectly impacting Spo0A activity to trigger sporulation initiation and transcription of these sigma factors; however, additional investigation into the molecular and genetic mechanisms underlying this phenotype is necessary.

Despite similar conclusions between these two studies, some differences in phenotypes were observed, which might help to hone future approaches for the mechanistic dissection of butyrate-dependent sporulation. For example, our work did not detect elevated spore counts during log phase growth (46), while Baldassare et al. did (63). In addition, our work was unable to show that butyrate increased spore counts using 70:30 medium (46), while Baldassare et al. did (63). These discrepancies could be due to differences in media types used (modified reinforced clostridial medium [mRCM] vs BHI) or differences in the methods of detection for the 70:30 medium. Both groups used the same strain and medium (70:30); however, this difference in spore quantification methods (phase contrast microscopy, which does not assay spore viability, vs culturing viable spores) may have led to the conflicting results, as spores must be germination-proficient to be detected via culturing. Regardless, the detection of increased sporulation by butyrate in BHI and mRCM, two media known for low sporulation efficiency, from two separate research groups, indicates the importance of butyrate-rich environments in initiating sporulation in *C. difficile*. Furthermore, this work highlights the utility of diverse *in vitro* growth conditions to define nodes within the complex regulatory network that controls *C. difficile* sporulation.

## DISTINCT PHENOTYPES OR AN INTEGRATED RESPONSE?

**Fig 1:**
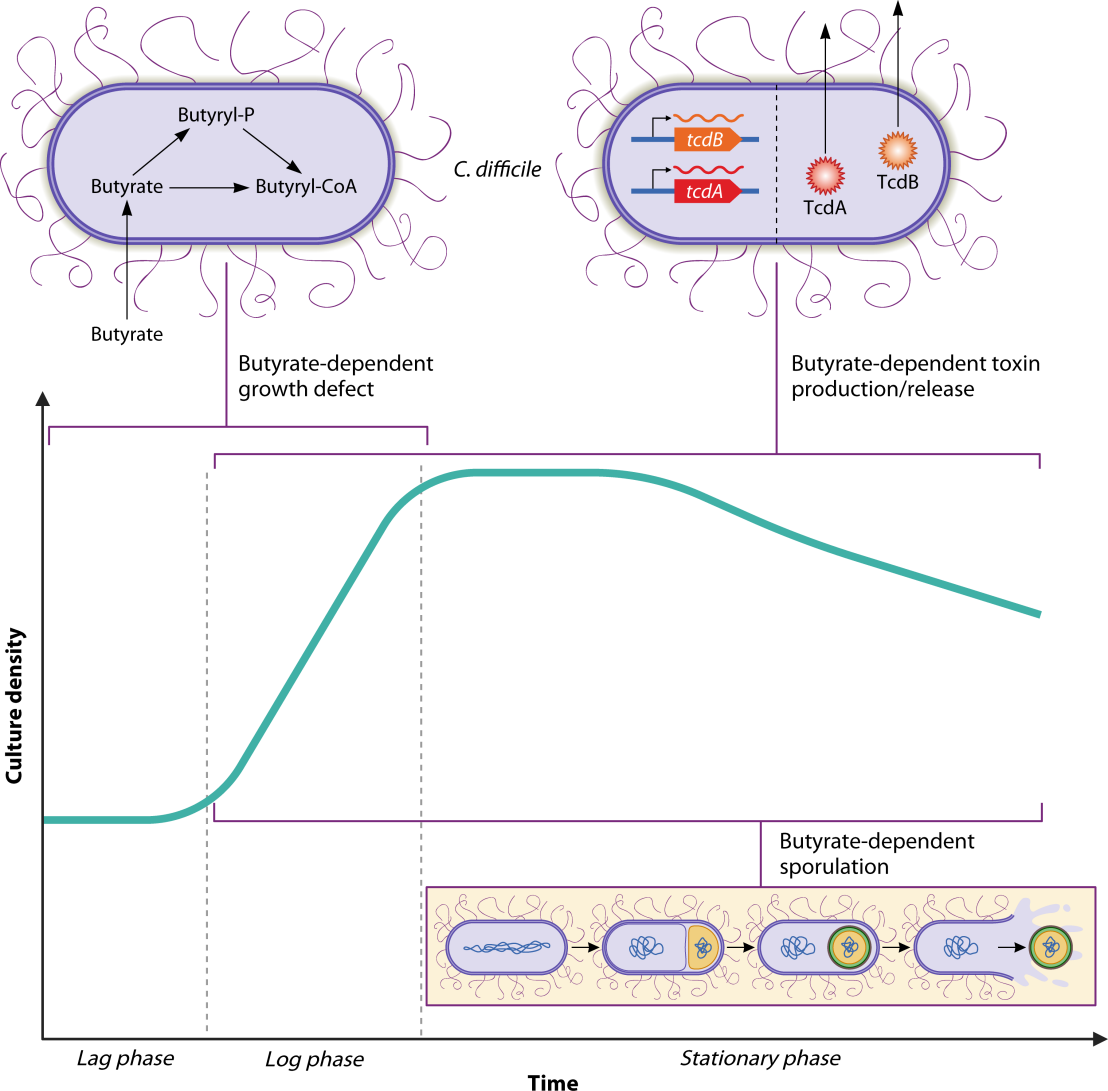
Timing of butyrate’s effects on *C. difficile* biology *in vitro*. Three butyrate-dependent phenotypes are observed in rich growth media. A butyrate-dependent growth defect is observed during log-phase growth, while both sporulation and toxin phenotypes are observed starting during log-phase growth and into stationary-phase growth, depending on experimental conditions. The specialized callouts highlight current understanding and existing gaps in knowledge of these phenotypes. The butyrate-dependent growth defect coincides with internalization of exogenous butyrate and accumulation of intracellular butyryl-CoA and downstream metabolic intermediates in *C. difficile*’s butyrate production pathways. Butyrate-dependent toxin gene expression and toxin release are likely involved in the toxin phenotype. Butyrate-dependent sporulation has been observed; however, little is known regarding how this occurs on a molecular level. Additional research is necessary to further unravel the mechanism(s) underlying all three of these phenotypes. Artwork courtesy of Patrick Lane, ScEYEnce Studios; reprinted with permission.

Butyrate exposure leads to three well-documented phenotypes in *C. difficile*: growth inhibition, increased toxin production/release, and enhanced sporulation. These phenotypes could reflect an integrated physiological response mediated by to-be-determined regulatory mechanisms. CodY and CcpA, which sense nutrient status, simultaneously repress toxin and sporulation genes in nutrient-rich environments (27, 29). RstA drives sporulation but dampens toxin expression and motility (82). If butyrate impacts the activity of one or more of these regulators, either directly or via intracellular metabolites (such as butyryl-CoA) that accumulate during butyrate exposure, it could lead to co-regulation of these phenotypes (Tables 1 to 3; Fig. 1). However, the known modes of sporulation and toxin co-regulation in *C. difficile* impact transcript levels of both *spo0A* and the toxin genes. Therefore, due to the absence of strong butyrate-dependent changes to *tcdA*, *tcdB*, and *spo0A* transcripts in existing studies, it is reasonable to hypothesize that these phenotypes are regulated by distinct and possibly novel mechanisms.

Alternatively, and in support of segregation of these phenotypes, *C. difficile* can partition into distinct sub-populations, where some cells express toxin, others sporulate, and a minority do both (83). The physical environment (e.g., solid vs liquid media) appears to reinforce this division of labor. In this context, butyrate may act as a differential signal to modulate behavior in a subset of cells based on local butyrate concentration, intracellular redox status, or proximity to other microbes. In line with this idea, the concentration of butyrate matters. Dose-dependent effects on butyrate-dependent phenotypes have been observed (Tables 1 to 3). While cecal and colonic butyrate levels can reach ~25 mM (49, 84), bulk measurements do not account for concentration gradients that exist due to the spatial organization of producers/consumers (85). A *C. difficile* cell adjacent to a strong butyrate-producing organism may experience a drastically different environment than one that is several microns or even millimeters away. So, while some previous work to uncover butyrate-dependent effects on *C. difficile* used “super-physiological” concentrations of butyrate, we feel that this choice is appropriate due to these limitations and as a tool to identify and dissect phenotypes, especially if dose-dependent effects are observed. So, the sum of these effects could have varying impacts on *C. difficile’s* responses to butyrate, especially if phenotypes are sensitive to different threshold concentrations. Taken together, there is an unmet need for further dissection of the molecular, genetic, metabolic, and ecological underpinnings of these phenotypes.

## POSSIBLE BENEFITS AND RISKS OF THERAPEUTIC MANIPULATION OF BUTYRATE DURING CDI

From a therapeutic standpoint, butyrate-dependent growth inhibition, butyrate-dependent modulation of the immune system, and observations that butyrate-rich gut environments exclude *C. difficile* are encouraging. This suggests that therapeutic elevation of butyrate in the GI tract could be useful in mitigating CDI in at-risk human populations. However, in addition to these effects, emerging evidence supports that butyrate is a cue exploited by *C. difficile* to time virulence and transmission. Butyrate-dependent elevation of toxins and spores suggests that *C. difficile* may sense rising butyrate levels as a signal of an inhospitable environment, where competing microbes are in abundance or one that is at carrying capacity for *C. difficile*. Either way, increased sporulation would enhance transmission to new hosts. Likewise, elevations in *C. difficile* toxins increase inflammation, which may slow host recovery, reduce the fitness of inflammation-sensitive microbes with which *C. difficile* competes for nutrients, and further aid in transmission. Butyrate-dependent effects on these virulence traits support the hypothesis that butyrate acts as a signaling molecule for *C. difficile*, akin to those used in quorum sensing.

In a complex host-associated microbial ecosystem like the gut, which of these butyrate-dependent effects is dominant? Previous work showed that switching mice from a fiber-deficient to a fiber-rich diet (characterized by low GI butyrate and high GI butyrate), *C. difficile* burdens are suppressed, but there is a transient increase in detectable toxins in the stool, which correlates with a transient increase in GI inflammation (37). In this case, it is reasonable to hypothesize that butyrate-dependent growth inhibition, butyrate-dependent effects on the host, and butyrate-independent effects of fiber dominate over the effects of toxin expression. However, as described above in section “The impacts of fiber consumption and GI butyrate on CDI in mice and humans,” there is emerging evidence that suggests that multiple variables can influence the balance of these effects.

**Fig 2:**
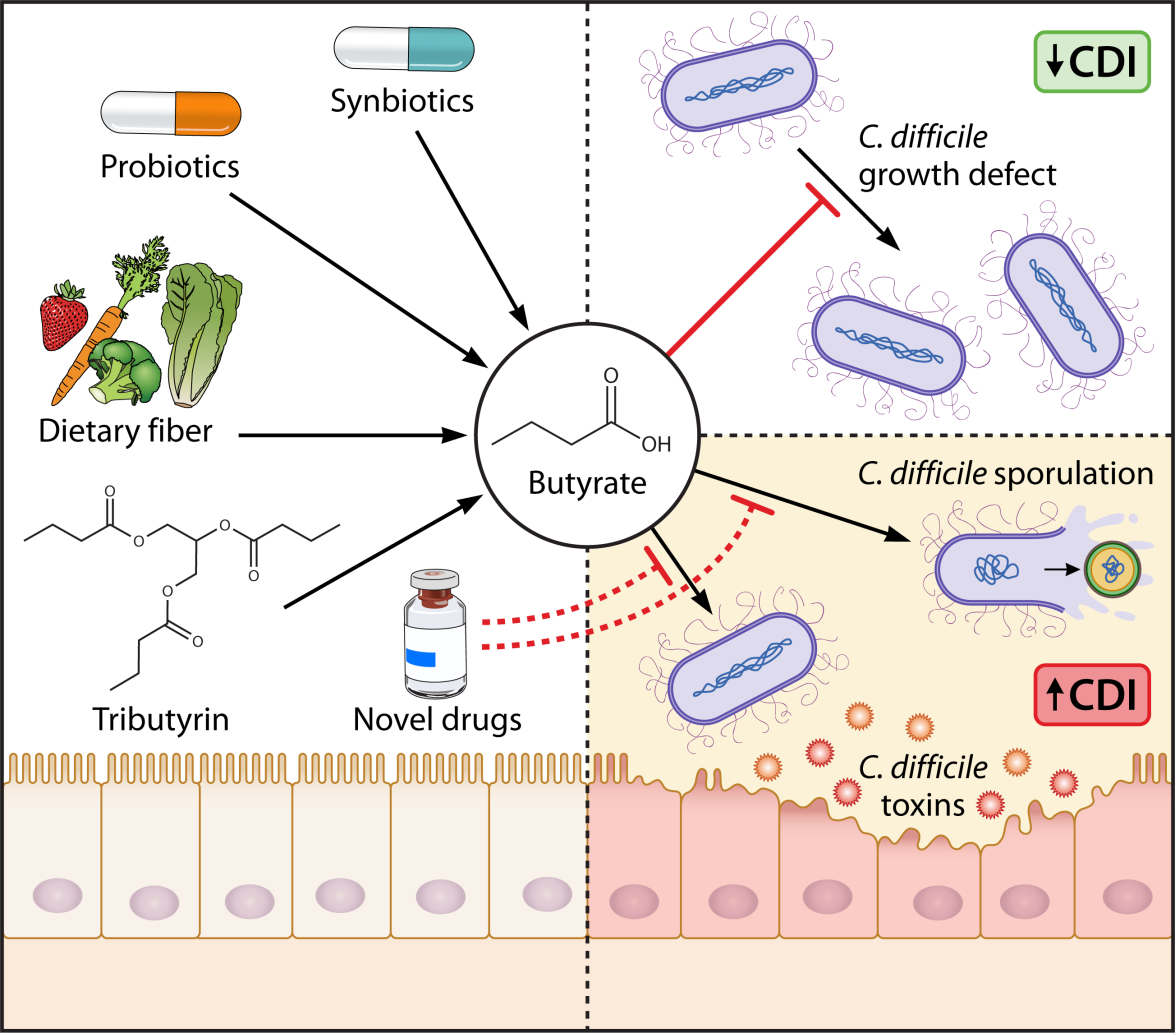
Possible therapeutic strategies to elevate gastrointestinal butyrate or target butyrate-responsive virulence traits. Possible strategies of elevating intestinal butyrate include dietary fiber, butyrogenic probiotics/synbiotics, and tributyrin administration. Elevated butyrate has the potential to reduce *C. difficile* burdens but also increase the expression of virulence traits. Therapeutic induction of the butyrate-induced growth defect could decrease *C. difficile* proliferation and simultaneously offer other gut microbes a competitive nutritional advantage against *C. difficile*. However, therapeutic elevation of butyrate could also positively impact butyrate-induced toxin expression/release and sporulation, which would further exacerbate CDI symptoms and promote transmission to new hosts. Additional research is needed to identify the host-, microbiome-, and *C. difficile*-based determinants that affect the balance of these phenotypes *in vivo,* which also has the potential to inform the development of new drugs to minimize toxin and sporulation phenotypes and favor CDI resolution. Artwork courtesy of Patrick Lane, ScEYEnce Studios; reprinted with permission.

Therefore, prior to translating these findings into therapeutic applications (Fig. 2), more basic research is needed. Fiber-based interventions are attractive because they are safe, accessible, and beneficial to host health. However, their success depends on microbiome composition, butyrate-independent effects of fiber (86–88), patient access to fiber-rich foods, and patient compliance. Synbiotics (the combination of probiotic organisms and prebiotics, non-digestible fibers which feed the probiotic organism) may overcome some of these complications and could be readily engineered based on known fiber-degrading and butyrate-producing probiotic organisms. Another option could be oral administration of butyrate or tributyrin. These strategies have shown efficacy in mice by protecting intestinal epithelial cells from *C. difficile* toxins through stabilization of HIF-1 (57). Alternatively, new drugs could be developed based on findings from ongoing research in this area. Specifically, small molecule inhibitors of key steps involved in butyrate-dependent regulation of toxins or sporulation could be developed, which could be administered to specifically inhibit butyrate-responsive virulence phenotypes.

## CONCLUSIONS AND FUTURE DIRECTIONS

Current data show that butyrate-rich gut environments are generally non-permissive to *C. difficile* colonization and disease. While butyrate negatively impacts *C. difficile* fitness *in vitro*, butyrate exposure also leads to elevations in *C. difficile* toxins and enhanced sporulation. These combined observations underscore the importance of butyrate as a modulator of gut ecosystem function and as a cue for *C. difficile* to regulate its virulence and transmission. While substantial progress has been made, critical questions remain. First, we lack a mechanistic understanding of whether butyrate’s growth inhibitory effects are a direct result of interference with intracellular CoA pools, altered redox balance, cytoplasmic acidification (89), or pushing butyrogenic reactions in an unfavorable direction. Comparisons of *C. difficile* with analogous literature on other organisms’ responses to SCFAs (75, 89) will help to provide additional future research directions, define clade-specific responses, and better understand how SCFAs may directly shape the microbiome in health and disease. Second, our work suggests that toxin release, rather than canonical transcriptional control of *tcdA* and *tcdB*, is the dominant toxin-related phenotype under butyrate exposure. Butyrate-dependent upregulation of genes like *tcdE* and *cwp19*, as well as increased autolysis, supports this view. However, it is unclear if toxin secretion and autolysis pathways act simultaneously or on different timescales or if these effects are driven by butyrate itself or downstream metabolites (e.g., butyryl-CoA). Third, butyrate exposure enhances *C. difficile* sporulation but without altering *spo0A* transcript levels. This suggests that novel regulatory pathways are also involved in this context. One possibility is that there is post-transcriptional or post-translational modification of key regulators like Spo0A, for example, through butyrylation (as has been observed in *C. acetobutylicum* [90]) or through Hfq-dependent sRNAs (91). Alternatively, butyrate may impact known global regulators or stress responses in unappreciated ways to affect sporulation. Additionally, understanding the concentrations of butyrate that represent the “threshold” for each of these phenotypes is an important piece of the puzzle that remains to be answered and will further contextualize these phenotypes in the complex microbial and metabolic milieus of the gut. All of these—including whether growth, sporulation, and toxin effects are coordinated through similar regulatory pathways, which of these effects dominate *in vivo*, and over what time frames—represent clear targets for future molecular and genetic dissection.

Taken together, this review highlights the utility of microbial metabolites as entry points to understanding pathogen biology. Continued efforts to understand how butyrate (and other host and microbiome-derived signals) is sensed by *C. difficile* will yield a better understanding of its pathogenesis and perhaps inform strategies to mitigate CDI with minimal impacts to our microbiomes.
